# Local electricity price effects of Bitcoin mining in renewable-rich energy systems

**DOI:** 10.1016/j.isci.2026.117584

**Published:** 2026-09-23

**Authors:** Maximilian Gill, Jona Stinner, Marcel Tyrell

**Affiliations:** 1 Department of Philosophy, Politics & Economics, Witten/Herdecke University, Alfred-Herrhausen-Straße 50, Witten, Germany; 2 Department of Physics, Friedrich-Alexander University Erlangen-Nuremberg, Freyeslebenstraße 1, Erlangen, Germany

**Keywords:** Bitcoin mining, electricity markets, demand flexibility, energy and sustainability

## Abstract

Load-shifting is increasingly important in renewable-rich energy systems. Bitcoin mining is often cited as a large, theoretically flexible load. Despite electricity consumption rivaling medium-sized industrial economies, the energy market behavior and impacts of Bitcoin miners remain largely unexplored. We exploit the large-scale relocation of Bitcoin mining to Texas, which became the world’s largest mining hub following China’s 2021 ban, to estimate its effects on local wholesale electricity prices. Combining a hand-collected dataset on mining facility locations with high-frequency wholesale price data, we identify price impacts using a DiD design. We find that miners select into renewable-rich, high-GDP per capita counties with initially lower electricity prices on average. Entry has no robust effect on daytime prices but raises nighttime prices by 19.9%. Price separation widens most in already-stressed hours. Miners may curtail under extreme scarcity, but do not shift load across ordinary price cycles to complement renewable generation.

## Highlights

• Dataset maps 112 operational US Bitcoin mining facilities by end-2024
• Miners preferentially locate in high-renewable, high-GDP counties
• Treated counties show a regular nighttime electricity price increase of 19.9%
• Price separation widens most in already-stressed hours

## Graphical abstract

**Figure:**
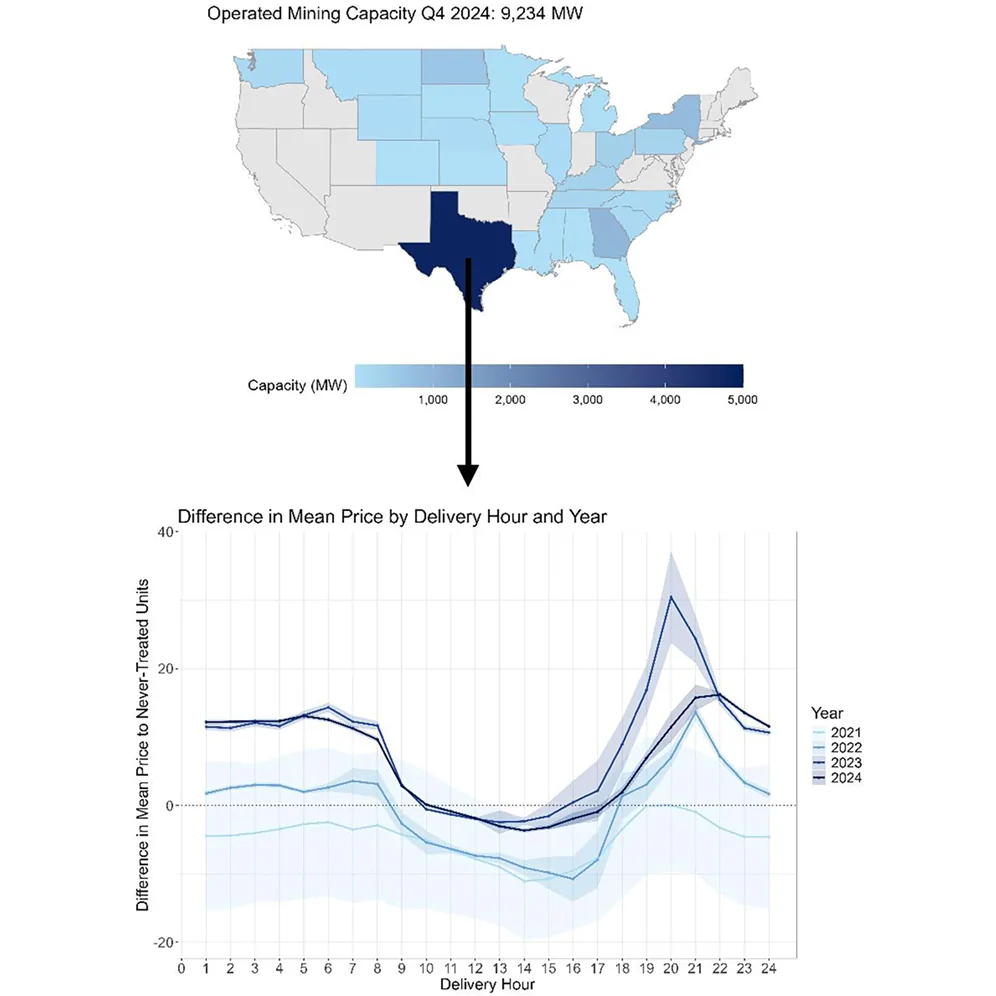

## Teaser

Applied sciences; Energy systems

## Introduction

Bitcoin mining, (we focus on Bitcoin mining because Bitcoin accounts for the largest share of market capitalization among proof-of-work blockchain networks; the conceptual arguments apply equally to other proof-of-work crypto-asset mining activities) with its substantial demand for electricity, has become a focal point in debates on climate and energy policy.^1^^,^^2^^,^^3^ The system’s electricity consumption is estimated at around 1% of global electricity demand, comparable to that of medium-sized countries such as Thailand or Spain.^4^^,^^5^^,^^6^^,^^7^

While critics stress the environmental externalities of Bitcoin mining, proponents argue that mining could support renewable investment by acting as a flexible demand source. Since mining operations can flexibly adjust electricity consumption, they may absorb surplus renewable generation that would otherwise be curtailed. Case evidence suggests that miners have indeed exploited excess hydropower and wind surpluses in China and the United States.^8^^,^^9^ By monetizing such curtailed resources, mining could raise returns on renewable projects and incentivize additional investment.^10^ However, the use of surplus electricity alone does not necessarily mitigate Bitcoin’s environmental externalities.^11^ A positive environmental impact through this mechanism would arise only if miners actually exercise their theoretical flexibility and shift loads to complement renewable generation. Two distinct readings of flexibility must be separated here. The first is the ability to curtail during genuine scarcity events or when compensated by system operators. Miners possess this technical interruptibility, and their price responsiveness at these extremes is well documented.^12^^,^^13^ The second is *load-shifting flexibility*: adjustment across ordinary price cycles, consuming when renewable output is abundant and withdrawing when the grid tightens. We analyze only the latter. It is the notion on which the environmental case rests, since raising demand in surplus hours would improve renewable project economics, while withdrawing it in tight hours is what would displace fossil generation.

Miners may be flexible in the first sense without being flexible in the second: if the price threshold at which mining becomes unprofitable lies above the range of ordinary price cycles, curtailment will occur only at the extremes, and miners therefore add demand in ordinary high-price hours as well.

Despite extensive debate on the environmental footprint of crypto-asset mining, empirical evidence on realized flexibility and its effects on electricity markets remains limited. Existing studies show that mining can raise average prices,^14^ but the effects on intraday timing and implications for investment incentives in generation and grid infrastructure remain scarce. A lack of reliable mining facility-level data has further constrained empirical analysis, leaving the market consequences of mining migration largely unexplored.

This study addresses this gap by analyzing how the recent influx of Bitcoin miners to the United States shapes electricity prices in Texas and, through these price effects, alters incentives for investment in generation and grid infrastructure.

Texas provides an ideal case for two reasons. First, the ban on crypto-asset mining in China in June 2021 triggered a large-scale relocation of miners, with a substantial share moving to the US. While China accounted for about 75% of global computing power in 2019 compared to only 4% in the US, the latter share rose to 35.5% shortly after the ban and is increasing until today.^5^ The installed mining capacity that we identify in this work accounts for approximately 69% of the global Bitcoin hashrate in early 2025, indicating a strong concentration of mining activity in the US (based on Antminer S21 specifications as the standard mining rig [234 TH/s at 0.00351 MW] and a 90% utilization rate [MARA, one of the largest mining companies report 99% uptime], the 9,234.5 MW installed capacity we identify correspond to ∼554 million TH/s, or ∼69% of the global hashrate [∼795 million TH/s on 1 January 2025, according to blockchain.com]). This is consistent with evidence reported by Makarov and Schoar^15^ of a sharp post-2022 increase in mining concentration that they attribute to the expansion of large-scale US mining. Texas emerged as the primary destination, hosting roughly half of US mining capacity, making it a natural laboratory for studying the market effects of mining activity.

Second, Texas operates one of the most market-driven electricity systems worldwide, with highly granular and responsive price signals. This allows to observe the effects of new demand sources in wholesale prices and subsequently to identify how miners influence local market dynamics.

For our analyses, we assembled a novel hand-collected dataset of all major US Bitcoin mining facilities. Because most operators are private and disclosure is limited, no official registry of mining activity exists. Our dataset, compiled from multiple independent sources in a multi-stage process, represents the first comprehensive mapping of Bitcoin mining activities until January 2025 in the US. We merge facility-level mining data with 15-min local marginal prices (LMPs) from ERCOT, Texas electricity system operator, to examine how price patterns evolve across counties with and without mining operations. To isolate the causal effect of miner entry, we employ difference-in-differences (DiDs) design suited to staggered adoption settings, estimating group-time average treatment effects following Callaway and Sant’Anna^16^ and Sun and Abraham.^17^ Our price analysis goes beyond average price effects by examining how mining influences prices across intraday periods and how these effects interact with fluctuations in renewable generation. In doing so, we assess whether miners provide flexibility or instead exacerbate system stress.

## Results

### Bitcoin mining migration

Lacking comprehensive data, we use a bottom-up approach and hand-collect facility data to reconstruct the migration of Bitcoin miners to the US, and to quantify their electricity demand. We identify 165 industrial-scale mining facilities. For 139 facilities, we observe commissioning dates, locations, and installed capacity. Of these, we validate 112 as operational at the end of the observation period, with an aggregate installed capacity of 9,234.5 MW. Considering the sector’s high opacity, our systematic investigation results in the most comprehensive publicly available dataset to date, providing a robust picture of Bitcoin mining activities in the US. For comparison, the US Energy Information Administration^18^ identifies 137 facilities with an estimated total capacity of 10,250 MW. These data are only available in aggregated form. The higher capacity estimate likely reflects reliance on company announcements, which in our site-level verification were often upward biased due to project delays, downsizing, or non-realization, unknown. The largest prior academic dataset^19^ identifies 34 facilities with an estimated capacity of 3,910 MW.

Figure 1 shows the development of operated Bitcoin mining capacities in the US between 2021 and 2024. Driven by the migration of miners from China to the US, capacities rose sharply between 2021 and 2022. The 9,234.5 MW operated at the end of our observation period is roughly equivalent to the electricity demand of 7.5 million average US households or 23.9 million average German households (the average demand of an US household according to the environmental impact assessment (EIA) is 10,791 kWh/year. The average demand of a German household reported by DESTATIS is 3,383 kWh/year. The energy capacity of the 9,234.5 MW at full load generates an electricity consumption of 80,894,220 MWh/year). In addition, 39 new facilities or expansions, amounting to 5,762.8 MW, were under construction and not yet reported operational by the end of our sample period in Q4 2024.

**Figure 1:**
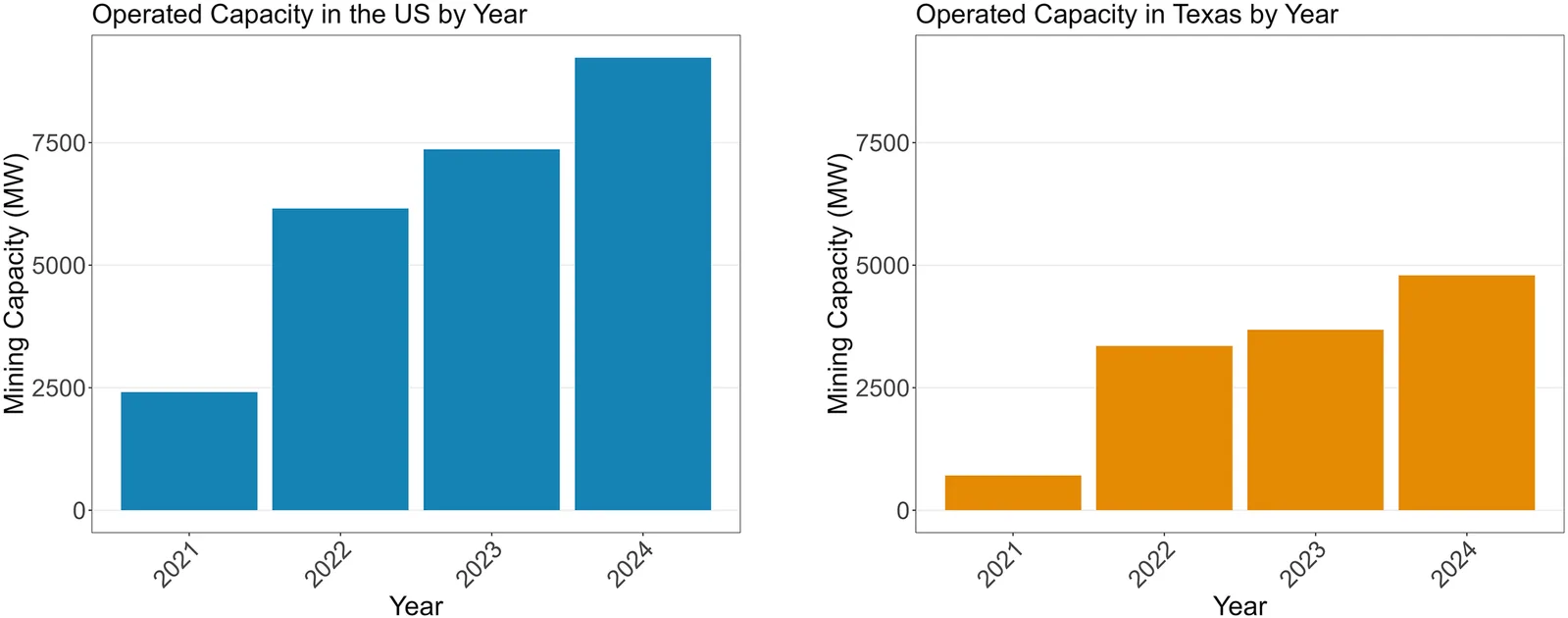
Operated mining capacity in the US and in Texas by year The figure shows the development of capacities in operation. We see a sharp increase in mining activity in 2022, after Bitcoin mining was banned in China in 2021.

Facility sizes vary widely. The smallest facility in the sample, located in Franklinton, North Carolina, operates at 0.5 MW, while the largest, in Rockdale, Texas, reaches 700 MW. The average capacity across all facilities is 82.5 MW. Table S1 shows an example of the five largest/smallest mining facilities in our sample. Figure 2 illustrates the geographic migration of crypto-mining activity to the US. By the end of 2024, over half of the nation’s mining capacity is concentrated in Texas, while many other states exhibit minimal or no activity. Further details are provided in Table S2.

**Figure 2:**
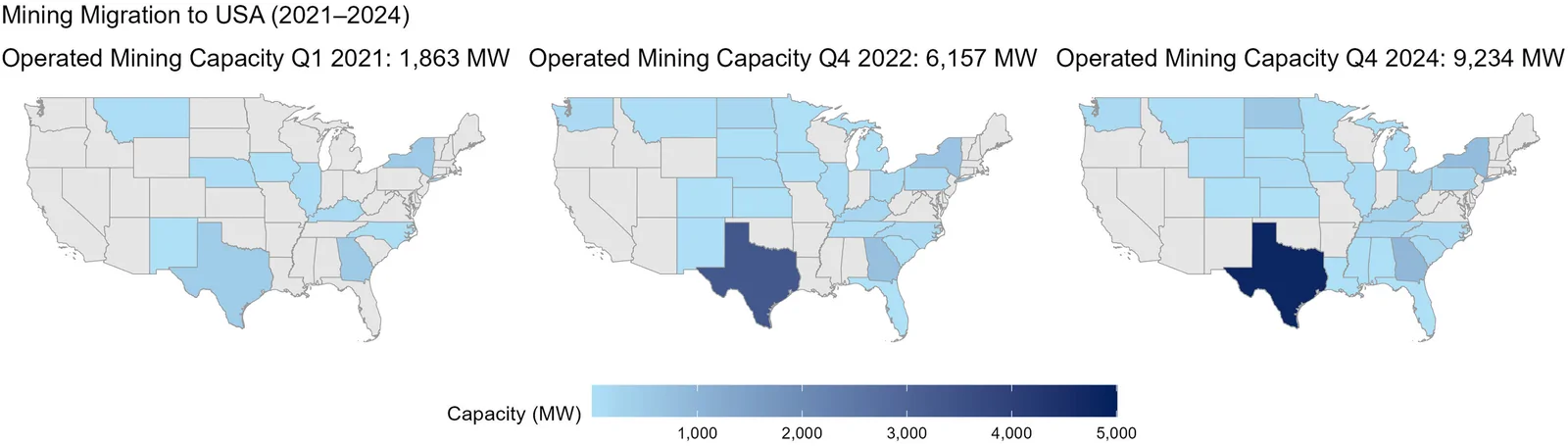
Map of operated mining capacities (Q1 2021, Q4 2022, and Q4 2024) The figure shows the expansion of 112 operational crypto-asset mining facilities in the US, totaling 9,234.5 MW, with a strong and growing concentration in Texas.

We study the entry decision of Bitcoin miners across US states using state-level data on electricity prices, energy capacity, industrial structure, and economic conditions. Only average industrial electricity prices significantly predict entry, identifying energy costs as the primary determinant of mining location choices.

Motivated by the strong spatial concentration of mining activity, we focus on Texas at the county level. In Q4 2024 we identify 30 Bitcoin mining facilities with an operated capacity of 4,789 MW in Texas. We geolocate these mining facilities at county-level and merge them with generator-level capacity from EIA Form 860.^20^ To examine county characteristics, we classify counties with active mining at the end of the sample period as treated and those without mining as untreated, and compare total electricity generation capacity, renewable shares, and gross domestic product (GDP) per capita across the two groups (we adopt different treatment definitions in this paper. End-of-sample classification is used for descriptive comparisons of county characteristics and trends, while treatment in the econometric analysis is defined at the facility commissioning date to identify causal effects of mining entry).

We find that miners locate in counties with higher average total capacity and a higher renewable share. By end-2024, untreated counties average 38% renewables, whereas treated counties exceed 66%. This pattern implies selection into more renewable-intensive counties relative to the average across Texas. Higher installed capacities in treated counties predate mining entry, and renewable capacity continues to expand more rapidly in these counties.

We augment our county dataset with GDP from the US Bureau of Economic Analysis, wholesale price data from ERCOT, and population data from the US Census Bureau, and compare treated and untreated counties (Figure 3). Miners disproportionately locate in counties that combine higher GDP per capita, a higher renewable share—especially solar (Figure 3D)—and a higher frequency of negative prices.

**Figure 3:**
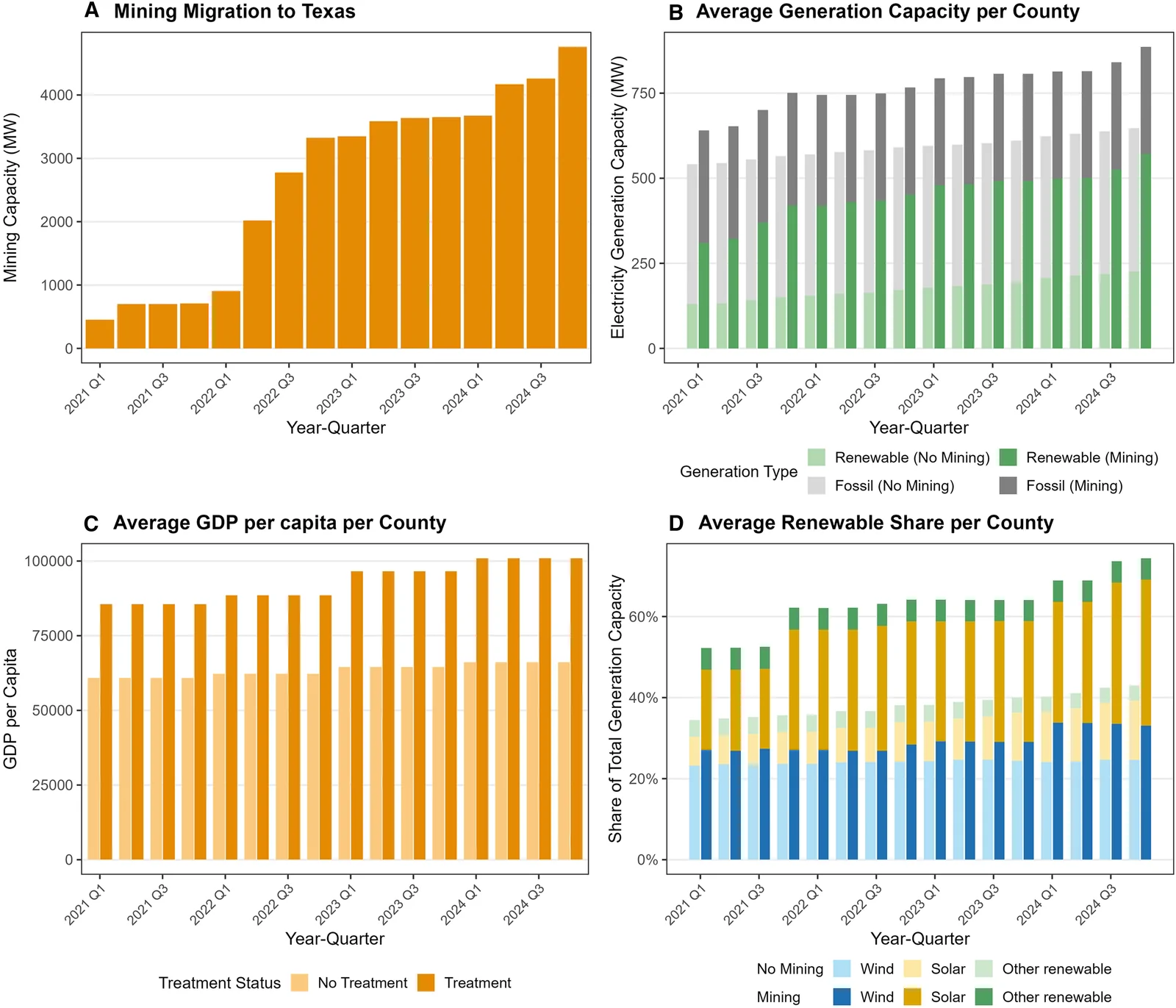
County-level comparison of treated (hosting Bitcoin mining facilities by end-2024) versus untreated counties in Texas (A) Operational Bitcoin mining capacity, with major increase in 2022. (B) Mean installed electricity generation capacity by source. The *Fossil* category aggregates all non-renewable generation, including nuclear. Treated counties had 21.4% higher total capacity in 2021, predating large-scale mining entry. (C) GDP per capita. Treated counties showed 24,727 USD higher GDP per capita in 2021, with faster growth through 2024. (D) Renewable share of installed capacity, by source (wind, solar, and other renewables). Mining facilities locate preferentially in counties with above average renewable penetration, and this gap widens over the observation period, driven by a rapid solar buildout in treated counties.

A high-GDP indicates selection into economically stronger, industrialized regions with existing energy infrastructure, where access to pre-built interconnections, substations, and transmission lowers entry and operating costs. An illustrative case is Milam County, where miners repurposed the former Alcoa aluminum facility: the site’s high-capacity substation and transmission access enabled rapid conversion to large-scale mining.^21^

A high frequency of negative prices, in turn, signals recurrent oversupply of (mostly renewable) generation. For miners, whose operating costs are dominated by electricity, this surplus is an attractive input, and treated counties exhibit it more often than untreated ones. The higher frequency of surplus is driven by wind at night and, especially, by solar during the day, reflecting the rapid expansion of solar capacity over the sample period.

The combination of reliable infrastructure and abundant low-cost power is a near-ideal setting for mining. That miners exploit such surplus is consistent with existing anecdotal evidence (Stinner and Tyrell^8^; Halaburda and Yermack^9^) and is the starting point of our analysis. Our findings indicate that they do so systematically in their choice of location. Whether they use this energy flexibly—curtailing when surplus disappears—is a distinct question we take up in the following section.

### Impact on energy prices in Texas

Next we examine how mining operations affect local electricity system conditions. We focus on intraday wholesale prices, which capture system stress and the marginal cost of supplying electricity. LMPs provide a direct measure of shifts in supply-demand dynamics and investment incentives. We therefore compare the evolution of LMPs in counties with Bitcoin mining activity to those without mining facilities. Bitcoin mining is often argued to support the energy transition because mining load is theoretically flexible and can shift consumption toward periods of abundant renewable generation.^10^ This claim is particularly relevant in Texas, where rapid solar expansion has increased the variability of both prices and CO_2_ intensity over the day. Because emissions intensities vary intraday, the timing of electricity demand is critical. Granular LMPs allow us to test whether mining activity aligns with low-price, low-emissions periods or reinforces demand during high-price, high-emissions hours.

Figure 4 documents the strong intraday variation in CO_2_ intensity, peaking during morning and evening demand surges and reaching its minimum during solar-rich midday hours. To capture these systematic differences in system conditions, we classify hours into daytime (08:00–19:59) and nighttime (00:00–07:59 and 20:00–23:59) in the subsequent analysis.

**Figure 4:**
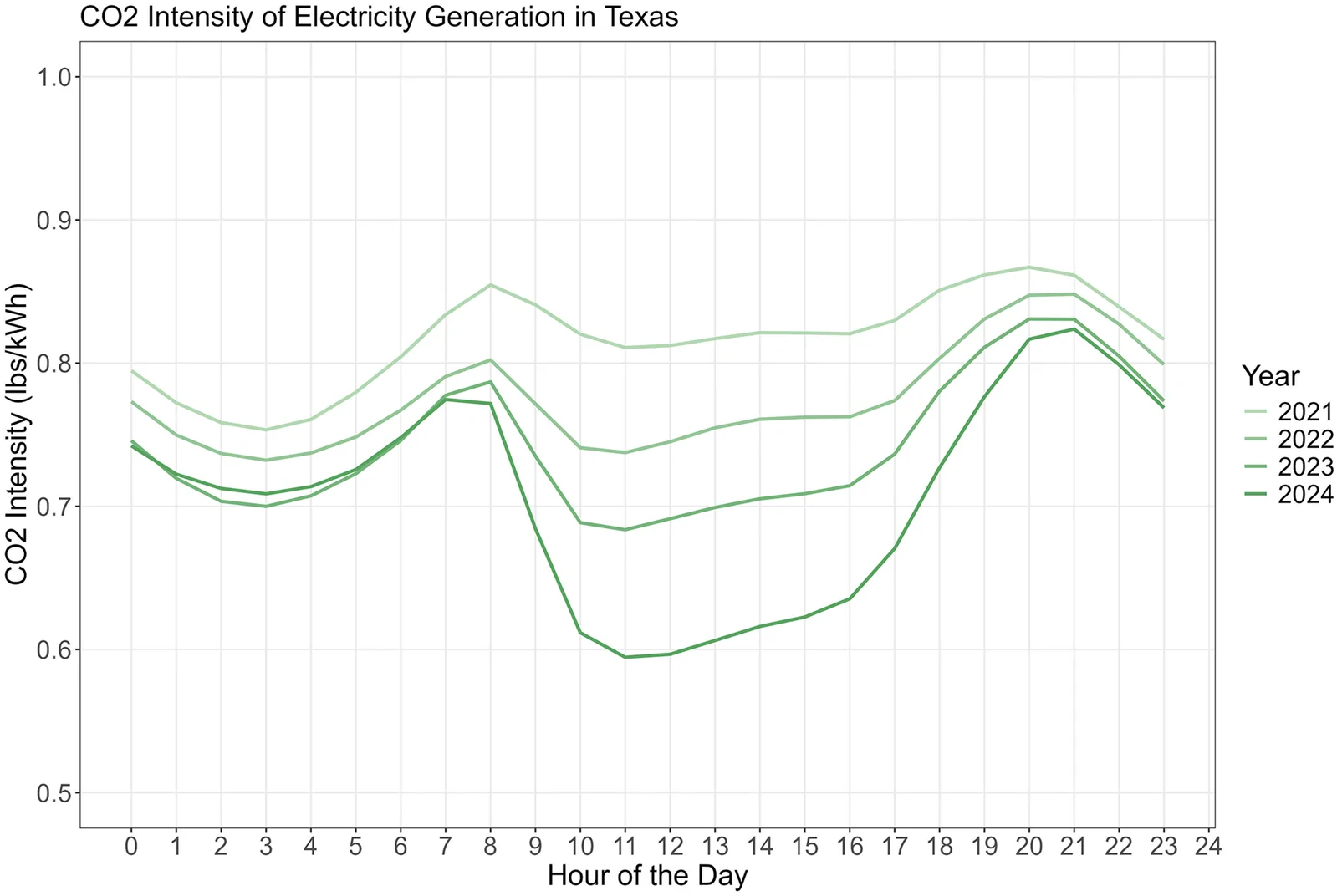
CO_2_ intensity of electricity generation in Texas The figure shows the average CO_2_ intensity of electricity generation in Texas over the course of the day during the observation period. CO_2_ intensity peaks in the morning and evening when system demand rises and peaker plants are dispatched. In contrast, intensity declines sharply around midday, reflecting the high share of solar generation. Nighttime CO_2_ intensity remains consistently higher than daytime levels. Data: EIA-930 Hourly Grid Monitor (Texas).^22^

Before turning to the econometric analysis, we first examine descriptive differences in local prices near Bitcoin mining facilities. We classify all settlement points (SPs) located in counties with observed mining activity during the study period as *treated* (T) and all others as *untreated* (C). We then compare average intraday price profiles across these two groups to assess whether locations with mining already exhibit systematically different price patterns over the course of the day. For each year *y* and each hour of the day *h*, we compute the difference in average nodal prices between treated and non-treated counties as

$$\Delta P_{y,h}={\bar{P}}_{T,y,h}-{\bar{P}}_{C,y,h},$$

where ${\bar{P}}_{g,y,h}$ denotes the mean electricity price in group *g* ∈ {*T*, *C*} at hour *h* in year *y*, with *T* and *C* indicating treated and untreated SP, respectively.

If the difference equals zero, prices do not differ between treated and untreated settlement points. Negative values indicate lower LMPs at settlement points that subsequently attract Bitcoin miners, compared to points that never host mining activity. Positive values indicate the opposite. Figure 5 shows these price differences over the course of the day.

**Figure 5:**
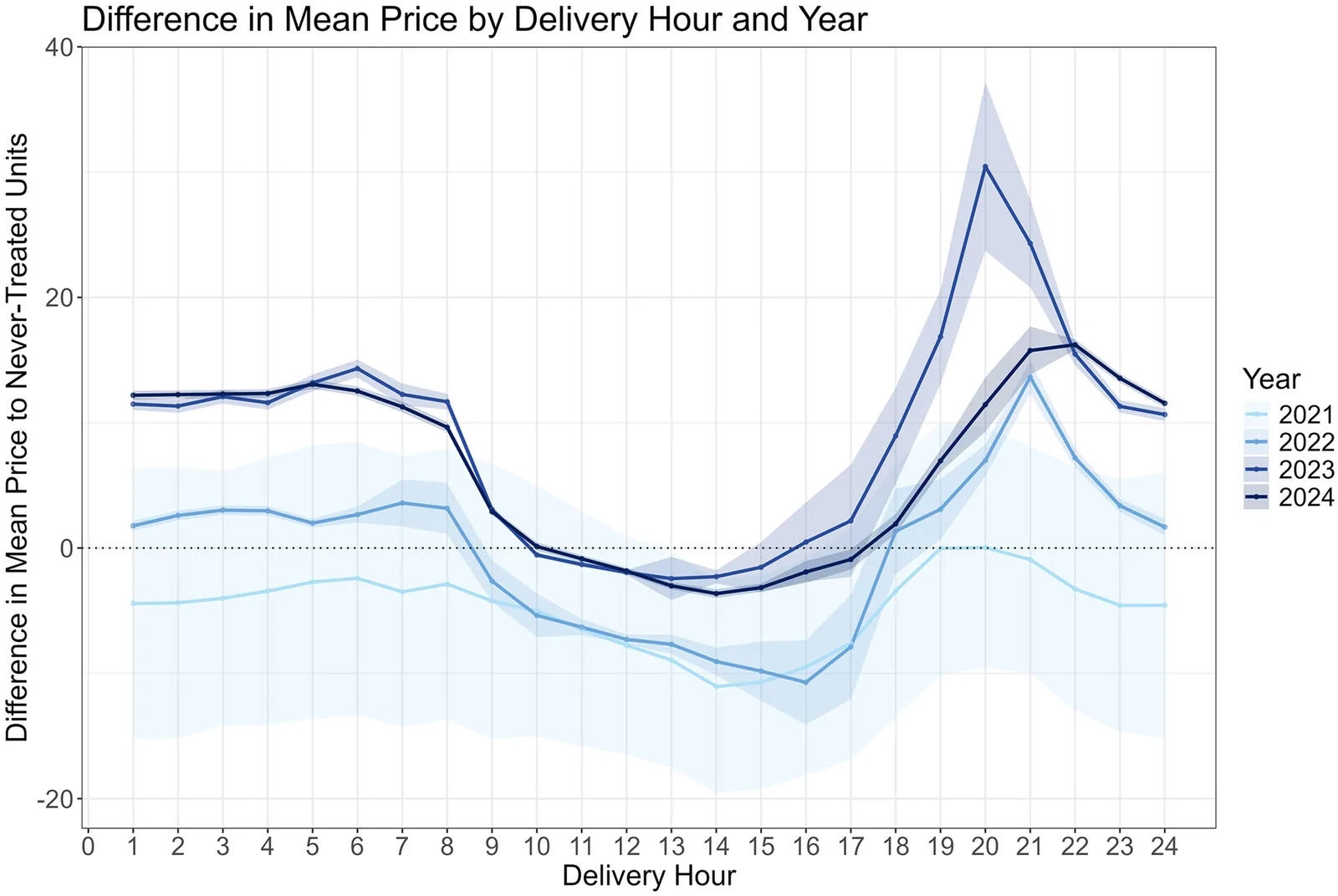
Hourly electricity price differences between mining and non-mining settlement points, 2021–2024 In 2021, settlement points that later become mining locations exhibit lower-or in the midday hours comparable-prices relative to untreated locations. Consistent with the economic logic of Bitcoin mining, operators appear to migrate to areas with systematically cheaper electricity to secure a competitive advantage. The resulting price differences display a duck-curve pattern, typical of regions with substantial solar penetration, which reflects the higher share of solar generation in these counties. Over the years, with rising mining activity, prices at treated settlement points rise relative to untreated ones. By 2023 and 2024, only the midday hours (10:00–15:00) remain cheaper, while most other hours become substantially more expensive. Bands are 95% confidence intervals of the difference in means. Note: the wide confidence interval in 2021 stems from Winter Storm Uri in Q1 2021, and the pronounced evening peak in 2023 is partly driven by the September 2023 heat wave; excluding these periods attenuates the respective effects. See Figure S2 for the same chart with these two periods excluded.

We find that Bitcoin miners settle in locations with initially lower average electricity prices, driven in particular by cheaper midday hours. As mining activity expands over time, prices at treated settlement points rise relative to untreated ones across the entire day. Our results show that daytime prices in 2023 and 2024 are similar to those in untreated counties. In contrast, nighttime prices become markedly more expensive.

We can see that price differences between treated and untreated counties evolve in tandem with the influx of miners to Texas, with the magnitude and direction varying by time of day. But how much of this pattern can be attributed directly to mining activity? We employ the Callaway and Sant’Anna DiD estimator^16^ to identify the causal effect of mining activity on LMP prices, accounting for treatment effect heterogeneity in staggered adoption settings. We verify robustness using the Sun and Abraham estimator.^17^ Additionally, to ensure our results are not driven by the later expansion of artificial intelligence (AI) data centers, we re-estimate the model on a sample ending in Q4 2023. The nighttime effect is essentially unchanged (ATT 8.48 USD/MWh; Table S7). Methodological details are provided in the STAR Methods section.

The graphical and numerical results for the first six-quarters after miners arrive are presented in Figure 6; Tables S3 and S4. During daytime hours, the estimated effect is slightly positive (3.04 USD/MWh), with no immediate response in the dynamics. This estimate is, however, not robust to relaxations of the parallel-trends assumption that we test with the sensitivity analysis of Rambachan and Roth.^23^ We show the results of this test in Figure S3. We therefore do not interpret the daytime estimate as a causal effect of mining entry. The rapid expansion of solar capacity seen in Figure 3D may attenuate the effect of the added demand.

**Figure 6:**
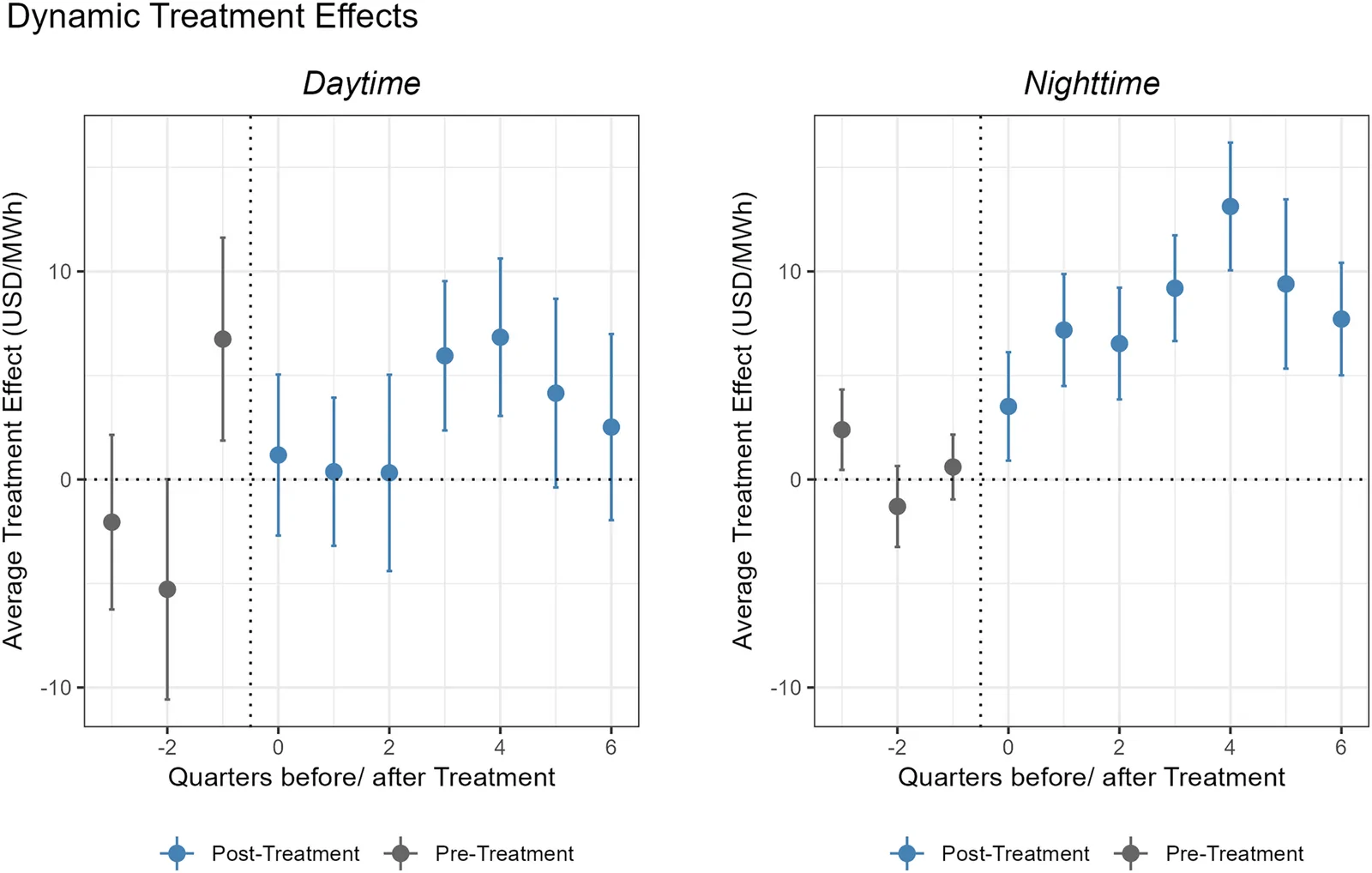
Callaway and Sant’Anna DiD estimates The dynamic treatment effects of Bitcoin mining entry on wholesale electricity prices, measured as average treatment effects on the treated (ATT) in USD/MWh relative to event time. Bands are 95% simultaneous (uniform) confidence bands. Pre-treatment coefficients are not uniformly zero but show no systematic pre-trends or anticipatory price responses, particularly during nighttime hours where post-treatment effects emerge. During daytime hours, mining entry does not generate statistically significant price changes in the first three post-treatment quarters. By contrast, nighttime prices increase sharply after treatment, rising over the first four-quarters and remaining persistently elevated thereafter. All post-treatment nighttime estimates are statistically significant, indicating a sustained demand-side price effect. The pronounced day-night asymmetry suggests that mining activity primarily increases price pressure during off-peak hours, when baseline demand is lower and marginal generation is more constrained.

In contrast, the nighttime effect is positive, statistically significant, and it remains significant under relaxations of the parallel-trends assumption. Such a trend is neither visible in the pre-treatment path nor supported by any evident change in the generation mix of treated counties, making the nighttime result robust. The estimated average treatment effect of Bitcoin mining is an increase of 8.1 USD per MWh, corresponding to 19.9% relative to the pre-treatment average price of 40.5 USD per MWh at nighttime in the treated counties.

A question is whether this nighttime price increase simply reflects miners absorbing otherwise-curtailed renewable generation. If so, the increase would be a consequence of clearing oversupply, and mining would be exercising load-shifting. This hypothesis makes two distributional predictions that we can test: mining entry should reduce the frequency of negative prices, and already tight high-price hours should be unaffected. We test both using the same event study design as for mean prices. Neither of the predictions holds at night. The frequency of negative-price hours is essentially unchanged after entry (Figures 7A and 7B), indicating that the surplus is not cleared, while the frequency of high-price hours (the top quartile of the within-period nighttime price distribution) rises by 8.1 percentage points, from a baseline of 32.8%. The nighttime price increase is thus not concentrated where power is cheap but extends into hours of system stress, a pattern that a load-shifting, surplus-absorbing flexible demand cannot produce and that is instead characteristic of an inflexible load (dynamics shown in Figure 8).

**Figure 7:**
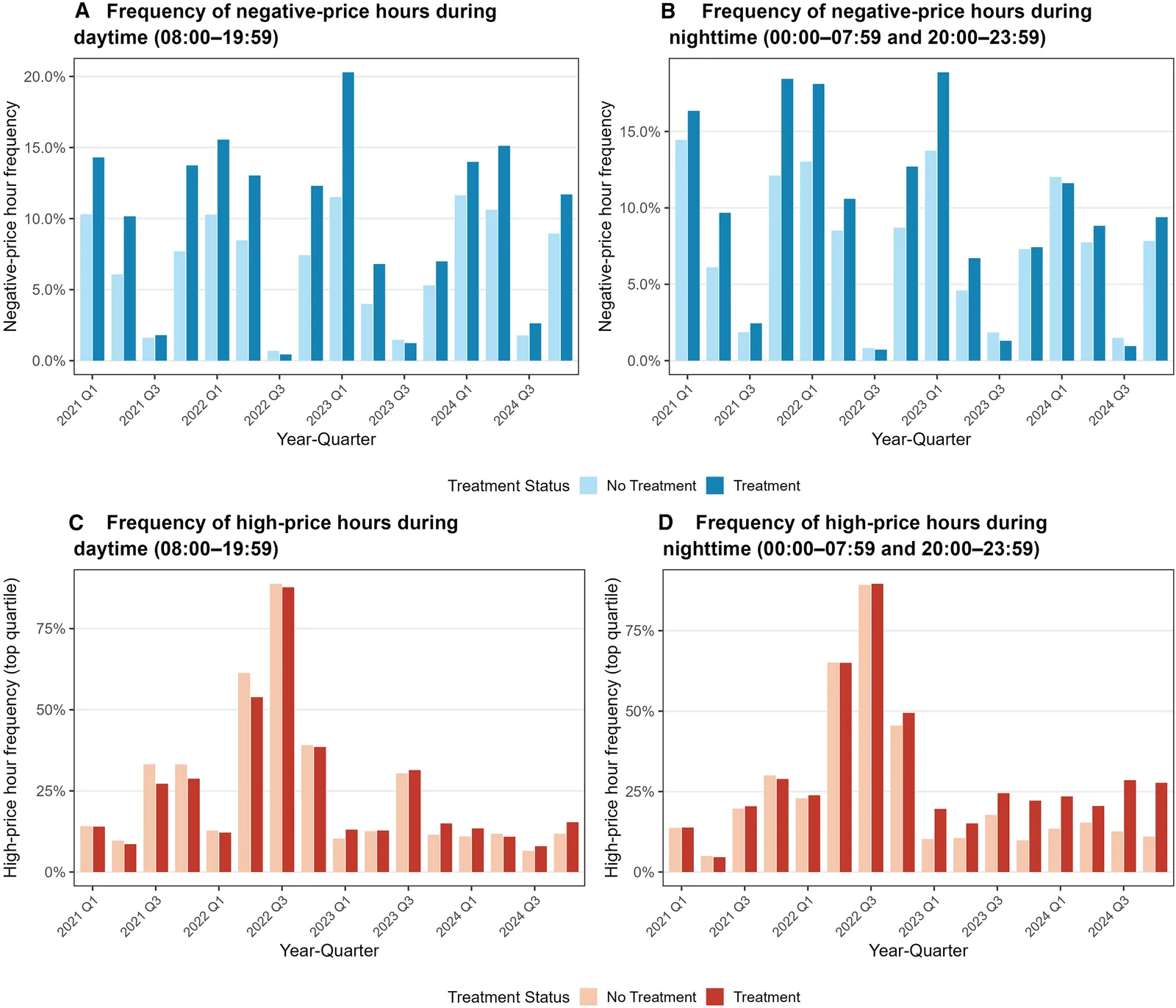
County-level comparison of treated (hosting Bitcoin mining facilities by end-2024) versus untreated counties in Texas regarding the frequency of negative and high-price hours (A) Negative-price hours occur more frequently in treated counties and remain present throughout the observation period. (B) Negative-price hours remain a recurrent feature of treated counties throughout the observation period and do not disappear over time. (C) High-price-hour frequency is similar in treated and untreated counties throughout the observation period. (D) Treated and untreated counties are similar early in the sample. From late 2022 onward, high-price hours occur more frequently in treated counties, and the gap widens notably over the remainder of the observation period.

**Figure 8:**
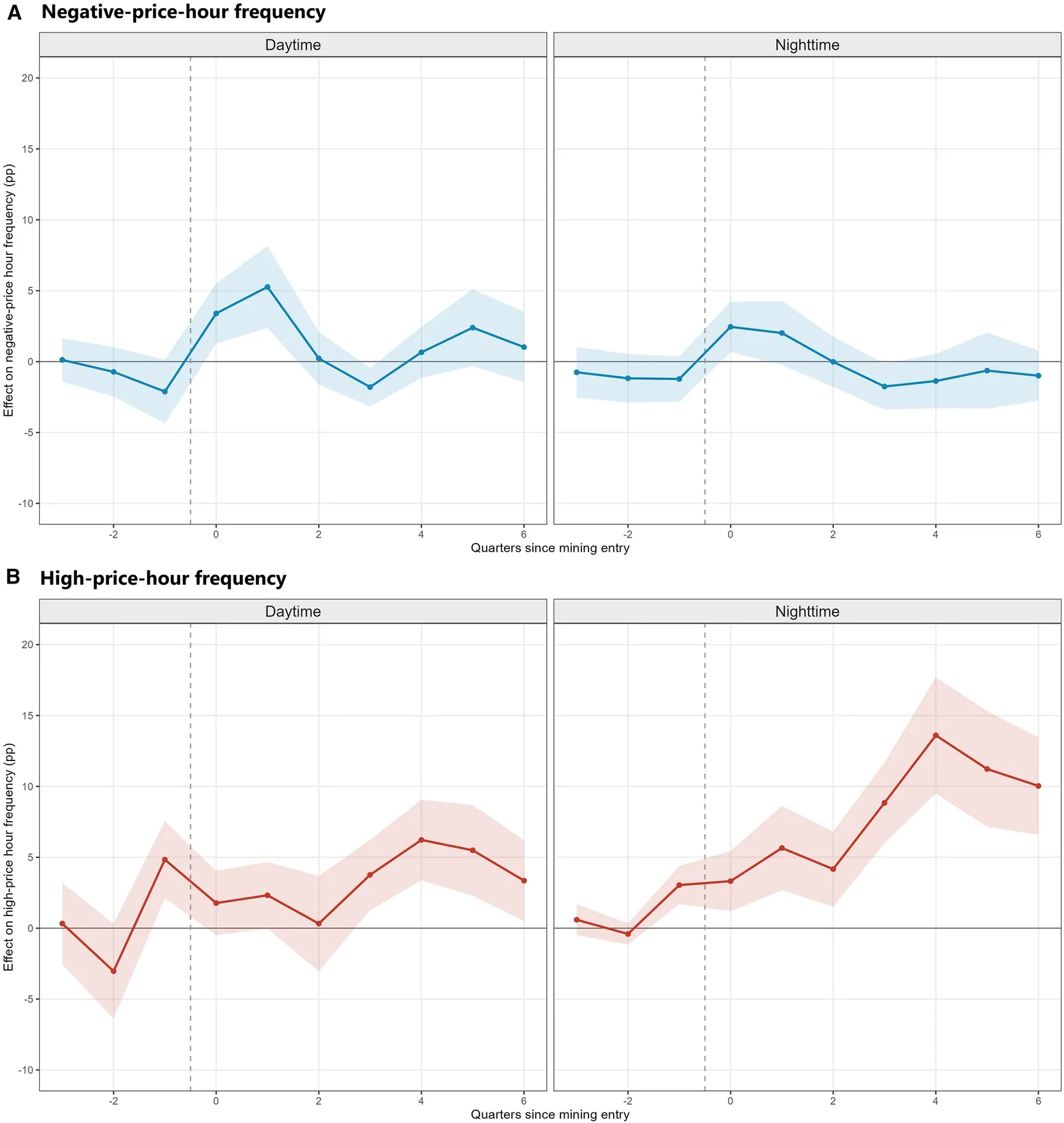
Effect of mining entry on negative and high-price-hour frequency (Callaway and Sant’Anna event study) Dynamic ATT estimates in percentage points on: (A) the frequency of hours with negative prices ( < 0 USD/MWh). Mining entry does not reduce the frequency of negative-price hours. Miners do not clear the existing surplus. (B) On the frequency of high-price hours (the top quartile of prices within each class and the sample period). The daytime effect is small and emerges only in later periods, whereas the nighttime effect is large, increasing, and persistent: mining entry raises the frequency of high-price, system-stress hours at night. Bands are 95% simultaneous (uniform) confidence bands.

Because prices differ across nodes only where transmission constraints bind, our estimates capture the localized component of mining’s price impact and should be read as a lower bound on the total effect. Excluding counties adjacent to mining activity from the control group leaves the nighttime estimate essentially unchanged (8.09 versus 8.32 USD/MWh; Tables S4 and S9), indicating that spillovers into neighboring counties are minor and, if anything, bias our estimates downward.

The mechanism behind this effect becomes visible when prices are examined separately for hours of system stress. Prices at two settlement points diverge only when transmission constraints bind, so localized price separation is a direct signature of congestion. We therefore compare the price gap between treated and untreated nodes in stress hours—the top quartile of prices within class and quarter, classified using untreated nodes only—against normal hours, before and after mining entry (Figure 9). Before mining entry, treated and untreated nodes price almost identically during normal nighttime hours (+0.1 USD/MWh) and separate only under stress (+7.3), reflecting the congestion-prone locations that miners select. After entry, both gaps widen, but the stress-hour gap widens far more: to +26.9 USD/MWh, against +7.6 in normal hours. This is descriptive evidence on where and when separation arises. Its implication is direct: A load providing everyday demand flexibility would reduce separation in stressed hours, curtailing precisely when congestion binds. We observe the opposite. After miners arrive, treated nodes separate from the rest of the market most sharply in exactly the stressed hours. This is the opposite of flexible behavior. These findings imply that miners migrate into counties with abundant renewable generation and frequent surplus, but that they use this surplus as any other large, inflexible load would. They profit from cheap electricity during hours of overproduction, yet they do not curtail once the surplus disappears. At night, and particularly during hours when the system is already stressed, their continued operation adds demand and increases local congestion. Continuous operation is privately optimal: it lowers average capital costs through higher equipment utilization, and miners curtail only when electricity prices rise so far that the marginal revenue from the next hash no longer covers them. That threshold lies above the high-price hours that occur routinely, so miners add demand in those hours as well. Within the ordinary operating range, the load-shaping benefits attributed to mining are therefore not evident in local prices, and added demand falls also into the hours when renewable output is lowest and CO_2_ intensity is highest.

**Figure 9:**
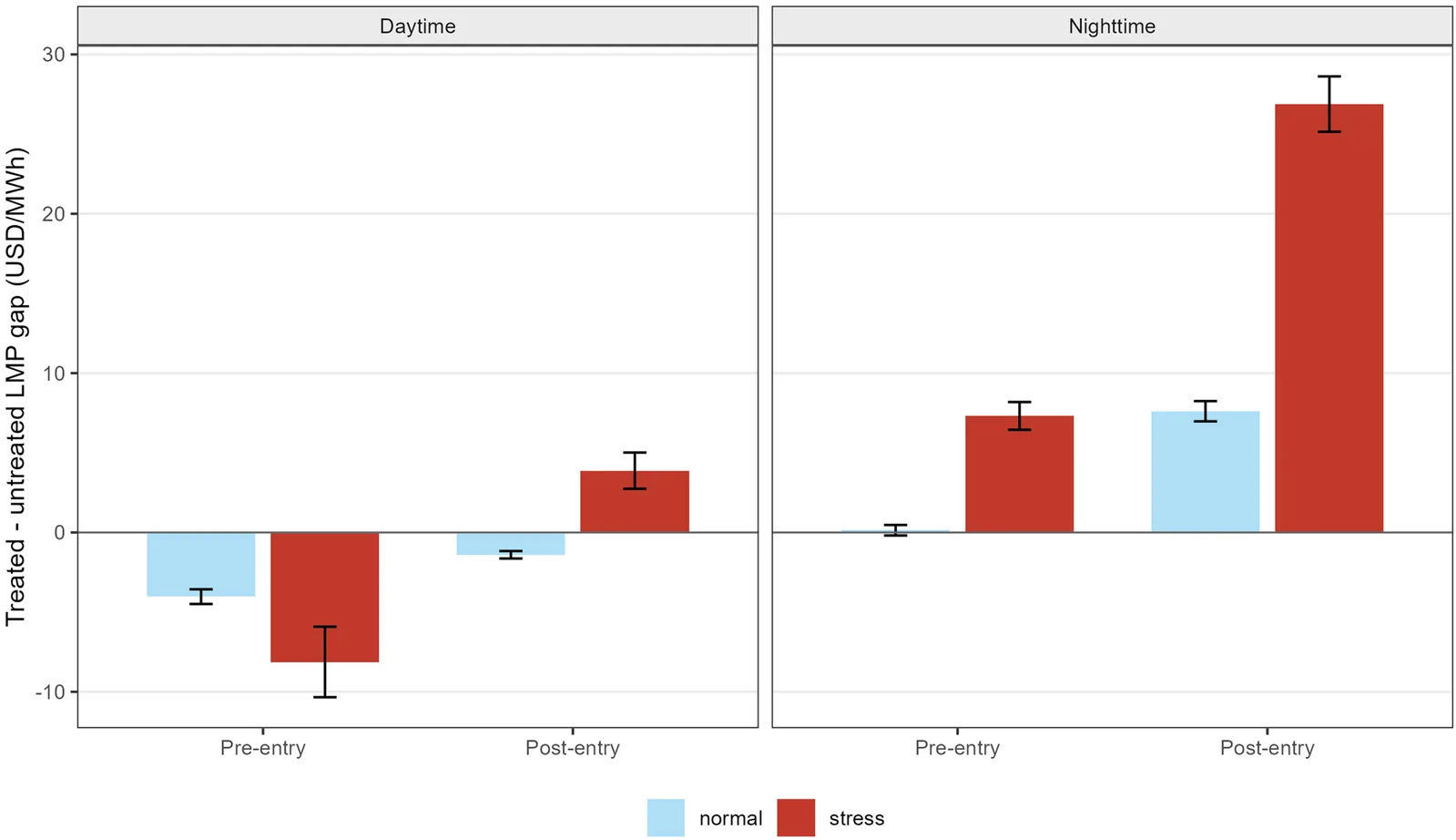
Treated-untreated price separation in stress and normal hours, before and after mining entry The figure shows the mean price gap between treated-county settlement points and the contemporaneous untreated mean, split by time of day, by mining status of the county, and by system condition. Error bars are 95% confidence intervals of the mean gap, computed across node–quarter cells. Stress hours are the top quartile of prices within class and quarter, classified using untreated nodes only, so that the classification is independent of the treated nodes’ own response. Winter Storm Uri (2021 Q1) and the September 2023 heat wave (2023 Q3) are excluded, and observations are aggregated to node-quarter cells. At night, treated and untreated nodes price almost identically in normal hours before entry (+0.1 USD/MWh) and separate only under stress (+7.3). After entry, the normal-hour gap rises to +7.6 while the stress-hour gap widens sharply to +26.9. Daytime stress hours reverse sign, from −8.1 USD/MWh before entry to +3.9 after.

## Discussion

Our results show a rapid relocation of Bitcoin mining from China to the United States following China’s ban, with a sharp increase in mining facilities in 2022. This expansion is highly concentrated geographically: Texas alone hosts roughly 50% of total US mining capacity. We attribute this clustering to cost minimization and a favorable regulatory environment. Low electricity prices are the primary driver, reinforced by a deregulated energy market, low regulatory barriers, and access to demand response programs, which jointly lower operating costs and uncertainty. Proponents and industry representatives claim that the influx benefits Texas by supplying demand-side flexibility to an electricity system with a high share of variable renewable generation.

An emerging body of literature examines the role of crypto-asset mining in facilitating the green energy transition.^24^^,^^25^^,^^26^^,^^27^^,^^28^ More specifically, proponents argue that mining could monetize otherwise curtailed renewable energy, improving the profitability of renewable projects. If this process accelerates renewable capacity expansion and displaces fossil-fueled power plants, Bitcoin mining could yield a net environmental benefit under appropriate electricity pricing policies,^10^ provided that the emission reductions outweigh the mining-related externalities.

Our results confirm that miners disproportionately locate in regions with a higher share of renewable generation. This implies that Bitcoin mining in Texas is supplied by a cleaner-than-average energy mix. However, higher renewable capacities predate miners’ entry.

Mining entry raises nighttime prices by 8.1 USD/MWh (19.9%) with no robust daytime effect. This is not the pattern a load-shifting, surplus-absorbing load would produce. A load-shifting demand would clear the surplus and leave tight hours untouched. Neither happens: negative-price hours remain as frequent as before entry, and high-price nighttime hours become more common. In stressed nighttime hours the treated-untreated price gap widens. Miners add demand where the system can least absorb it.

This is privately optimal. Capital utilization dominates the marginal electricity cost, so miners run continuously, curtailing only when prices exceed the marginal revenue of a hash or when they are paid to shut down events that do not recur with the renewable production cycle. What follows is this: renewable producers gain from monetizing otherwise curtailed output, demand stays high in fossil-intensive hours, and other consumers pay more, most of all when the grid is already tight. The influx of miners does not ease the system; it stresses it further.

Future research should examine whether the establishment of Bitcoin mining facilities affects long-term investment patterns in electricity systems. While prices respond relatively quickly to demand shocks, investments in generation, storage, and grid infrastructure are characterized by long planning horizons and adjustment delays. These investments shape future system configurations and thus the long-run consequences of additional electricity demand. An open question is whether mining activity induces additional renewable energy investment and, if so, whether this expansion displaces fossil generation or merely adds capacity on top of the existing mix. A distinct and equally important question concerns investment incentives in complementary infrastructure. By creating a direct source of demand for surplus electricity, Bitcoin mining may reduce the relative profitability of investments in storage technologies and grid reinforcement, potentially generating opportunity costs for long-term decarbonization pathways. Understanding these investment trade-offs is crucial for evaluating the long-run system-level effects of the new electricity demand by Bitcoin miners in the context of a planned green energy transition.

### Limitations of the study

Price coverage, though dense, is incomplete for some counties with identified mining activity. Of the 21 Texas counties hosting industrial-scale mining during the observation period, 16 enter the estimation sample. Cass and Walker have no ERCOT settlement point in the data, and Midland none with a sufficiently complete price series; relaxing the balanced-panel requirement recovers Navarro but leaves these three counties outside the sample. Milam County, site of the Riot Rockdale facility, the largest operation in our data, has been operational since Q4 2019 and is therefore always treated relative to our observation period.

ERCOT offers near-ideal conditions for this question (nodal pricing, a large renewable share, and a concentrated influx of mining load) but energy systems in general, and ERCOT in particular, are complex, with demand and supply shifting simultaneously. AI data centers followed the miners into Texas and continue to enter; intermittent capacity, especially solar, has expanded rapidly; and extreme weather stresses the grid, as in Winter Storm Uri and the September 2023 heat wave. Disentangling these contemporaneous developments from mining entry is not straightforward. We therefore do not interpret the daytime estimate as causal: its robustness across specifications is insufficient to support that reading.

The DiD design identifies a relative, not a system-wide, effect. In a nodal market, prices diverge across settlement points only when transmission constraints bind, so any component of the price impact common to treated and untreated counties is absorbed. We observe the congestion-driven, locally visible portion, and our estimates are a lower bound on the total price impact of mining.

## Resource availability

### Lead contact

Requests for further information and resources should be directed to and will be fulfilled by the lead contact, Maximilian Gill (maximilian.gill@uni-wh.de).

### Materials availability

This study did not generate new unique reagents or materials.

### Data and code availability

• Data necessary to replicate the results in the study are provided in: https://doi.org/10.5281/zenodo.21597533.
• Code necessary to replicate the results in the study is provided in: https://doi.org/10.5281/zenodo.21597533.
• Any additional information required to analyze the data reported in this study is available from the lead contact upon request.

## Acknowledgments

We thank Lukas Stötzer, conference participants and discussants at the 2025 DGF Annual Meeting, the 8th Lindau Nobel Meeting in Economic Sciences, and the 2024 ISCFS for helpful comments; and Jeremy Westphal for excellent research assistance.

Parts of this paper were previously circulated under the title “Economic Limits of Bitcoin’s Environmental Promises: Pathway or Pitfall for the Green Transformation.” This study and our companion study, “Bitcoin’s Productivity Trap,”^11^ supersede those drafts.

## Author contributions

M.G.: conceptualization, methodology, software, validation, formal analysis, investigation, data curation, writing – original draft, visualization; J.S.: conceptualization, methodology, software, validation, formal analysis, investigation, data curation, writing – original draft, visualization; M.T.: conceptualization, methodology, validation, formal analysis, investigation, data curation, writing – review and editing.

## Declaration of interests

The authors declare no competing interests.

## Declaration of generative AI and AI-assisted technologies in the writing process

During the preparation of this work, the authors used Claude (Anthropic), ChatGPT (OpenAI), and Perplexity in order to identify mining facilities, as coding and debugging assistance, and to improve readability and language of the manuscript. After using these tools, the authors reviewed and edited the content as needed and take full responsibility for the content of the published article.

## STAR★Methods

### Key resources table

**Table:** 

REAGENT or RESOURCE	SOURCE	IDENTIFIER
**Deposited data**		

Dataset of U.S. industrial-scale Bitcoin mining facilities	This paper	Zenodo: https://doi.org/10.5281/zenodo.21597533
Replication code	This paper	Zenodo: https://doi.org/10.5281/zenodo.21597533
ERCOT 15-min Locational Marginal Prices, report NP6-905-CD	Electric Reliability Council of Texas (ERCOT)	https://www.ercot.com/mp/data-products/data-product-details?id=NP6-905-CD
ERCOT monthly Capacity, Demand and Reserves (CDR) Reports, used to map settlement points to Texas counties	Electric Reliability Council of Texas (ERCOT)	https://www.ercot.com/gridinfo/resource
Preliminary Monthly Electric Generator Inventory, Form EIA-860M	U.S. Energy Information Administration	https://www.eia.gov/electricity/data/eia860m/
Hourly Electric Grid Monitor, Form EIA-930, Texas	U.S. Energy Information Administration	https://www.eia.gov/electricity/gridmonitor/
County-level population estimates, Texas	U.S. Census Bureau	https://www.census.gov/data/tables/time-series/demo/popest/2020s-counties-total.html
County-level GDP, chained dollars	U.S. Bureau of Economic Analysis	https://www.bea.gov/sites/default/files/2024-12/lagdp1224.pdf
TIGER/Line cartographic boundary files, Texas counties (2023 vintage), used for county mapping and rook adjacency	U.S. Census Bureau	https://www.census.gov/geographies/mapping-files/time-series/geo/cartographic-boundary.html
SEC EDGAR company filings	U.S. Securities and Exchange Commission	https://www.sec.gov/edgar
Nexis Uni news, company and legal database	LexisNexis	https://www.lexisnexis.com/en-us/products/nexis-uni.page

**Software and algorithms**		

R, version 4.4.1	R Core Team	https://www.r-project.org/
sessionInfo.txt (record of the R version and all package versions used)	This paper	Zenodo: https://doi.org/10.5281/zenodo.21597533
did (Callaway–Sant’Anna group-time average treatment effects; main DiD estimator)	Callaway et al.^16^	https://CRAN.R-project.org/package=did
fixest (Sun–Abraham event-study estimator, sunab())	Sun et al.^17^	https://CRAN.R-project.org/package=fixest
HonestDiD (sensitivity of the DiD estimates to violations of parallel trends)	Rambachanet et al.^23^	https://github.com/asheshrambachan/HonestDiD
sf (spatial data handling, county geometries and adjacency)	CRAN R-Project	https://CRAN.R-project.org/package=sf
tigris (retrieval of U.S. Census TIGER/Line county shapefiles)	CRAN R-Project	https://CRAN.R-project.org/package=tigris

### Method details

#### Bitcoin mining data

Our first data set comprises hand-collected information on all major mining facilities in the United States. Crypto-asset mining facilities are operated by private operators with only a small share of publicly listed firms that disclose operation figures. Mining is the process of solving the SHA-256 hashing algorithm by brute force. Miners use specialized hardware, so called application-specific integrated circuits (ASICs), that are optimized solely for this computation and incapable of performing any other task. A facility identified with the business purpose “Bitcoin mining” therefore operates this function exclusively, allowing us to rule out any conflation with AI data centers.

In an attempt to provide a foundation for regulatory oversight, the EIA requested mining companies to share demand information, but the project was discontinued in early 2024 and no facility-level data was made available.^18^ Given this lack of a public registry of Bitcoin mining companies, we manually collected information from various sources in a multi-stage approach:

First, we compiled a list of Bitcoin mining companies operating in the U.S. from an extensive web-search. This search covered present and historical company websites and forum discussions from search-engine results and AI-supported tools using keywords like “crypto mining,” “Bitcoin mining,” “U.S.” We comprehended and verified our initial findings with industry reports, membership lists from interest groups (e.g., Crypto Mining Council, Texas Blockchain Council, The Miner Mag, Ensun), and available academic studies.^9^^,^^29^ Finally, we reviewed the EDGAR database of the U.S. Securities and Exchange Commission (SEC) for relevant company filings.

Second, we gathered operating capacity (MW), location, and the date of commissioning for each specific facility operated by the identified companies. We sourced this information from company websites, investor reports, presentations, and carefully verified it using the Nexis news database. (Nexis Uni contains data from over 17,000 news, company and legal sources.) To provide a solid (conservative) estimate, we only included facilities whose commissioning was confirmed. Announced projects without clear evidence of actual operation were excluded.

In the final step, we conducted targeted search requests and AI-based queries for each U.S. state (e.g., “Is there bitcoin mining in [state]?”). Additional locations identified through this process were added to the dataset, with, however, small overall volume and capacity.

#### Texas energy price data

We employ high-frequency LMPs from ERCOT to measure spatially and temporally granular variation in wholesale electricity market conditions. LMPs capture the marginal cost of serving an additional unit of electricity at a specific node (settlement point), making them a suitable proxy for localized supply conditions, congestion patterns, and system stress.

We obtain wholesale electricity prices from ERCOT’s *NP6-905-CD* report. ERCOT operates the Texas power grid and administers the state’s wholesale electricity market. We assign each ERCOT settlement point to a county using monthly *Capacity, Demand, and Reserve Reports*, allowing us to construct a county-level panel of wholesale electricity prices that we merge with mining facility locations and commissioning dates. The price dataset consists of 15-minute LMPs for 649 settlement points with price data for the whole observation period, covering 126 Texas counties and 16 of the 21 counties with active Bitcoin mining during the study period.(Five treated counties fall outside the estimation sample. Cass and Walker have no ERCOT settlement point in the LMP data; Milam, Navarro, and Midland have settlement points but none with a complete price series across all 16 quarters. Milam is additionally always treated, with mining operational from Q4 2019, and is therefore dropped by the DiD estimator regardless of price data availability. Relaxing the coverage requirement to 15 quarters using an unbalanced panels allows Navarro to be included into the analysis. This leaves the estimates essentially unchanged (see Tables S10 and S11.) To focus on systematic intraday price patterns rather than short-term volatility, we aggregate prices to hourly averages.

### Quantification and statistical analysis

#### Difference in differences

We employ a staggered-adoption DiD design to estimate the causal effect of Bitcoin mining activity on electricity prices. The analysis exploits temporal and spatial variation in the timing of mining adoption across Texas counties. Dynamic treatment effects are estimated using modern DiD methods designed for staggered adoption, with the estimator of Callaway and Sant’Anna^16^ as the primary specification and the event-study estimator of Sun and Abraham^17^ used for testing robustness of the results. To capture systematic differences in system conditions over the day, we classify hourly observations into daytime (08:00–19:59) and nighttime (00:00-07:59 and 20:00–23:59). The unit of observation is the ERCOT settlement point, which we assign to counties based on geographic location; outcomes are measured at the settlement point–quarter level and separately by time-of-day class, yielding a panel structured at the settlement point × quarter × class level. The sample period spans Q1 2021 to Q4 2024, covering 16 consecutive quarters. Treatment is defined as the presence of operational Bitcoin mining capacity in a county. Adoption occurs at different points in time across counties, generating a staggered treatment pattern. We estimate the average treatment effect of Bitcoin-mining adoption on quarterly average electricity prices at the settlement-point level. We cannot include county-level controls in our primary estimation using Callaway–Sant’Anna estimates because treatment cohorts are defined at the county level and often contain very few units, leaving no meaningful within-cohort variation in these variables.

#### Robustness checks

In the Sun–Abraham event-study specification, we additionally control for county–quarter characteristics on both the demand side (GDP and population) and the supply side (wind share, solar share, and total fossil capacity). Separating wind and solar, rather than using a single renewable aggregate, accounts for their opposing intraday profiles in ERCOT, where wind generation is higher at night and solar is concentrated during the day. The nighttime estimate is very similar to the baseline (ATT 8.39 USD/MWh), indicating that differential renewable buildout and fossil crowd-out do not account for the result. The daytime estimate is small and statistically insignificant in this specification (ATT 2.40 USD/MWh), consistent with our finding that the daytime effect is not robust. Full results are reported in Tables S5 and S6, and the method comparison in Figure S1.

Our main specification requires settlement points to have a complete price series across all 16 quarters, since the Callaway–Sant’Anna estimator is applied to a balanced panel. This excludes treated counties, most notably Navarro, which miss one single quarter of price data. Relaxing the requirement to 15 quarters in an unbalanced panel admits Navarro (400 MW of operated mining capacity) and additional settlement points, increasing the sample from 649 to 676. All other specification choices are unchanged. As Tables S10 and S11 show, the nighttime effect is marginally smaller but essentially unchanged (7.90 versus 8.09 USD/MWh), with the same dynamic path; the daytime estimate is likewise stable (2.98 versus 3.04).

Identification relies on a parallel-trends assumption, requiring that, absent Bitcoin mining adoption, electricity prices in treated and untreated settlement points would have evolved similarly. Each specification contains one significant pre-treatment coefficient (period −1 for daytime, period −3 at nighttime), but neither exhibits a systematic pre-trend. The deviations are isolated and alternate in sign. To assess sensitivity to violations of parallel trends directly, we apply the approach of Rambachan & Roth.^23^ The daytime estimate is not robust and we do not interpret it causally, whereas the nighttime estimate remains significant under relaxations of the assumption (see Figure S3). Such a trend would be difficult to rationalize economically: our Sun–Abraham specification with time-varying county controls yields very similar estimates, and the coefficient on fossil capacity is insignificant at night (see Table S6).
