# Supplementary material for: Local electricity price effects of Bitcoin mining in renewable-rich energy systems
Source: iScience. 2026 Sep 23;29(10):117584. doi: 10.1016/j.isci.2026.117584 (PMC13634397; doi:10.1016/j.isci.2026.117584)
Supplement: Supplementary file 1 [file mmc1.pdf]

iScience, Volume *29*

Supplemental information

Local electricity price effects of Bitcoin mining in renewable-rich energy systems

Maximilian Gill, Jona Stinner, and Marcel Tyrell

Supplemental Information

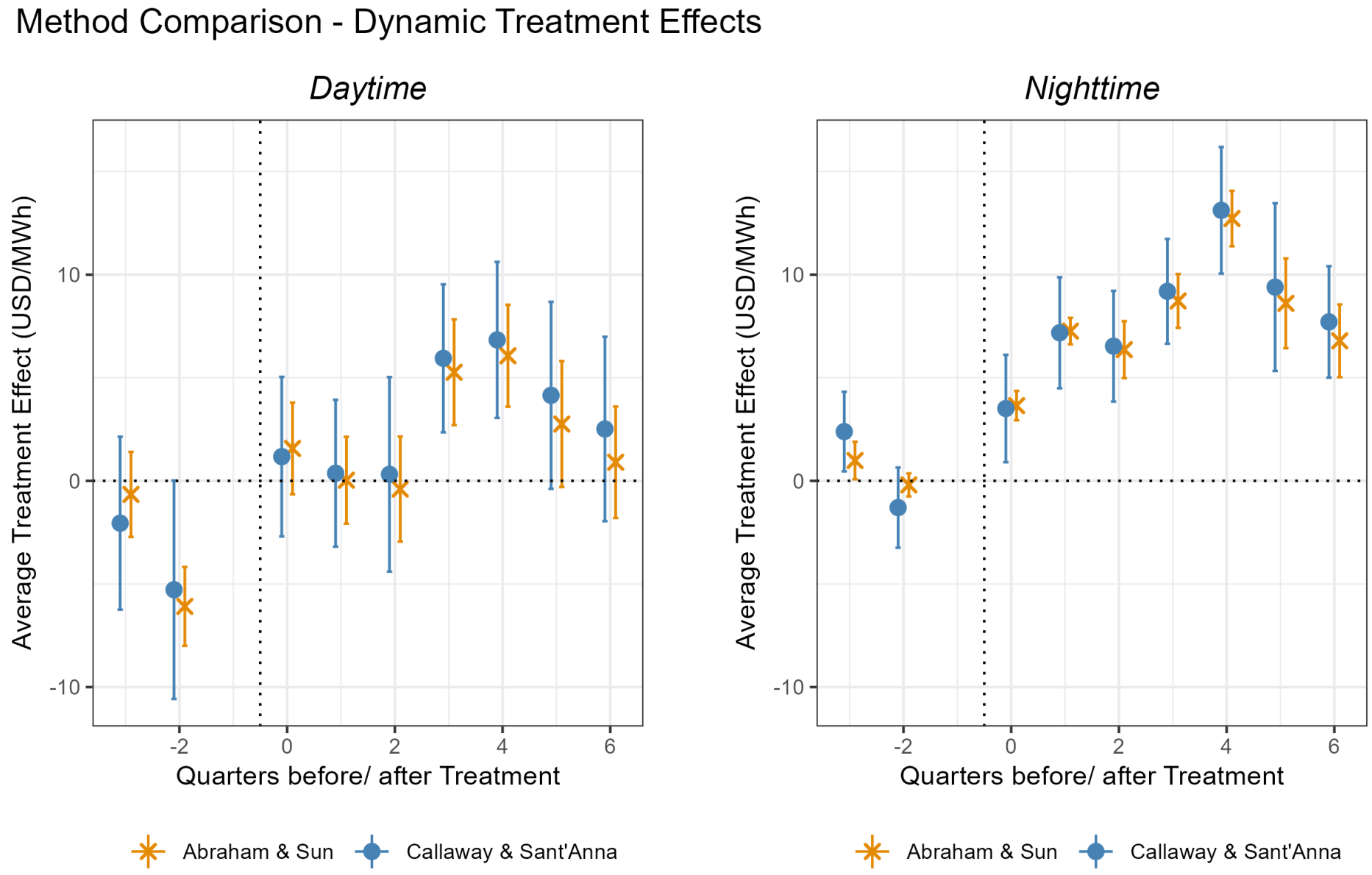

Fig. S1: Event Study Plot (Method Comparison)

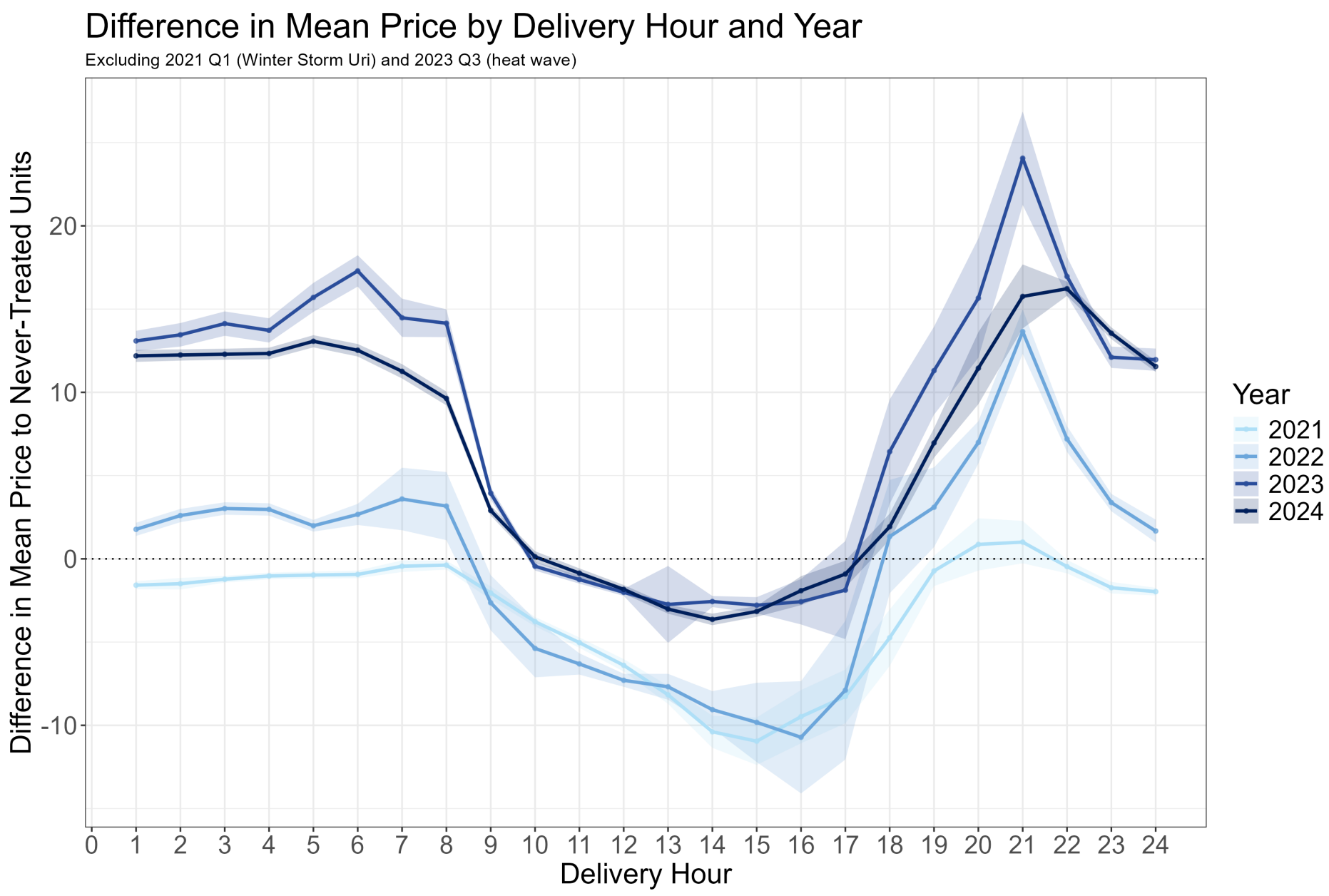

Fig. S2: Hourly Electricity Price Differences between Mining and Non-mining Settlement Points, 2021–2024; excluding Q1 2021 and Q3 2023)

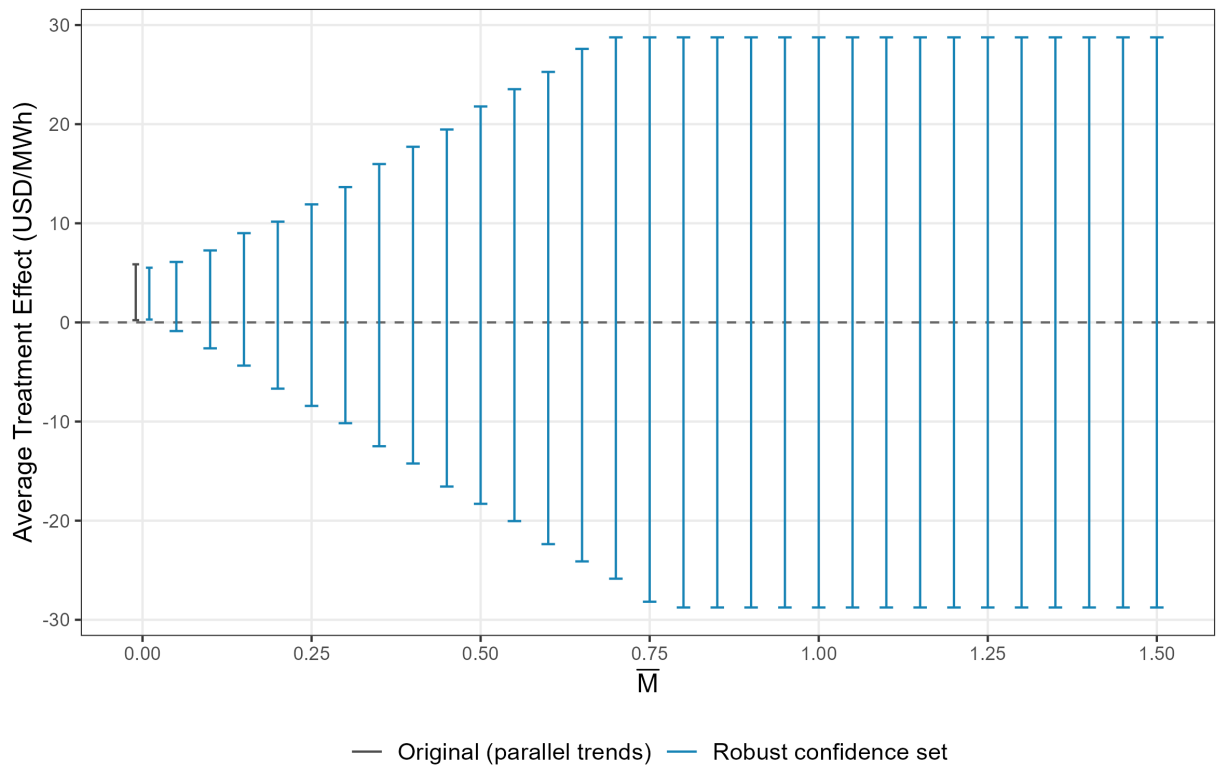

(a) Daytime

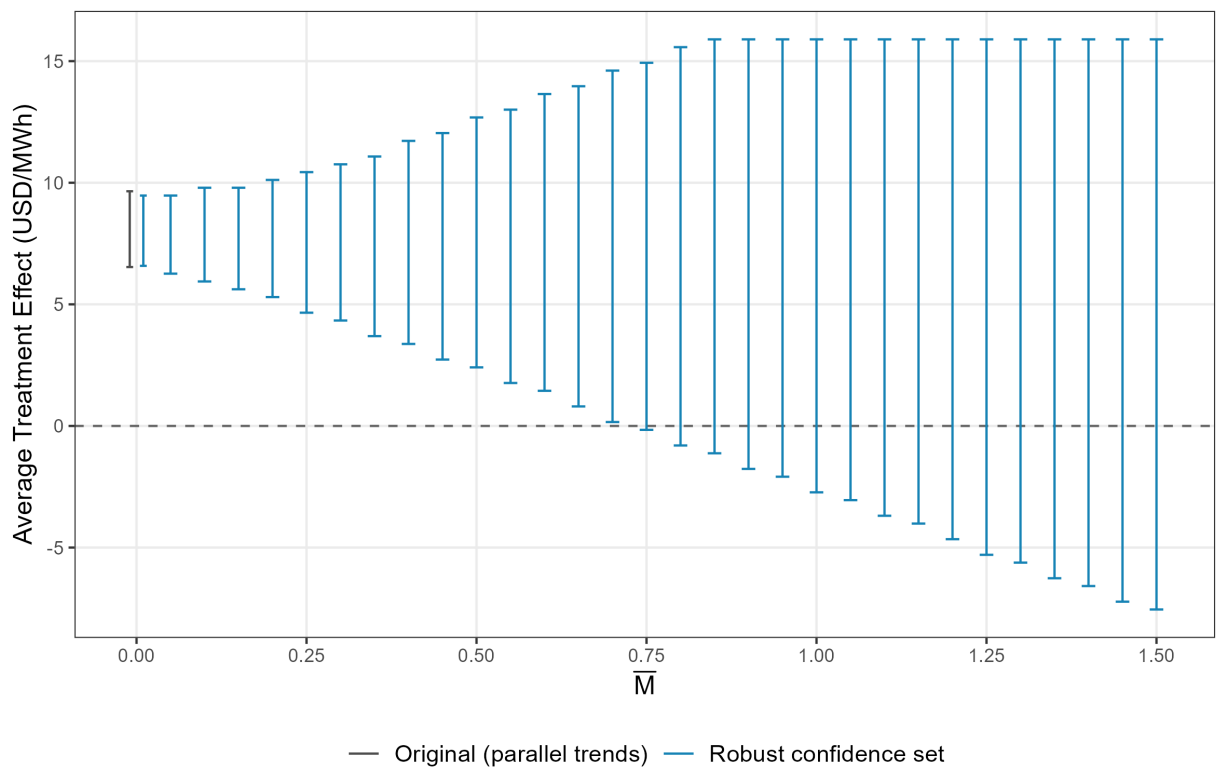

(b) Nighttime

Fig. S3: Sensitivity of the price effects to violations of parallel trends (Rambachan & Roth). Each panel plots the 95% robust confidence set for the average treatment effect as the permitted violation of parallel trends increases. M=0 is the original estimate under exact parallel trends. Increasing M allows the post-treatment differential to be a continued differential trend of M times the largest deviation in the pre-treatment period. The "breakdown value" is the M value at which the robust set first contains zero. Larger values indicate greater robustness. (a) The estimate is only significant under exact parallel trends assumption. The daytime effect does not survive even a minimal relaxation of the assumption, and we therefore do not interpret it as causal. (b) The robust confidence set excludes zero up to M = 0.7. The nighttime effect remains significant even when the counterfactual differential trend is allowed to be as large as roughly three-quarters of the largest deviation observed before entry. Note: Estimates use the universal base period required by the sensitivity analysis and therefore differ slightly from Tables S3 and S4.

**Company**	**State**	**City**	**Capacity (MW)**
Riot	Texas	Rockdale	700
Bitdeer	Texas	Rockdale	563
Coinmint	New York	Massena	435
Riot	Texas	Corsicana	400
Irin Energy Ltd.	Texas	Helios	350
⋮	⋮	⋮	⋮
Colorado Mining Companies LLLC	Colorado	not specified	3
Gryphon Digital Mining, Inc.	Louisiana	not specified	2.9
Bitzero Blockchain Inc.	North Dakota	Nekoma	2.5
Product Recovery Technology International (PRTI)	North Carolina	Franklinton	0.5
Acehost	Florida	Tampa	0.5

Table S1: Example of Mining Facilities in the Sample

State	Q4 2024		Q4 2022		Q1 2021	
	**Facilities**	**Capacity (MW)**	**Facilities**	**Capacity (MW)**	**Facilities**	**Capacity (MW)**
Texas	30	4789.0	22	3352.0	1	450.0
Georgia	10	931.0	9	716.0	3	485.0
New York	9	822.7	8	642.7	5	487.7
North Dakota	6	590.0	2	200.0	0	0.0
Ohio	5	383.0	2	141.0	0	0.0
Kentucky	9	294.0	6	218.0	1	125.0
Pennsylvania	5	278.8	3	172.0	0	0.0
Tennessee	5	228.0	2	32.0	0	0.0
South Carolina	6	148.0	2	55.0	0	0.0
Washington	3	142.0	2	129.0	0	0.0
North Carolina	4	133.5	4	133.5	3	55.5
Nebraska	1	100.0	1	100.0	1	100.0
Illinois	1	82.0	1	82.0	1	82.0
Montana	1	75.0	1	75.0	1	64.0
Wyoming	2	75.0	0	0.0	0	0.0
Mississippi	1	32.5	0	0.0	0	0.0
Alabama	1	28.0	0	0.0	0	0.0
Michigan	1	28.0	1	28.0	0	0.0
Iowa	4	20.6	3	15.6	1	5.6
Minnesota	1	20.0	1	20.0	0	0.0
Kansas	1	12.0	1	12.0	0	0.0
Colorado	2	9.0	2	9.0	0	0.0
South Dakota	1	6.0	1	6.0	0	0.0
Florida	2	3.5	2	3.5	0	0.0
Louisiana	1	2.9	0	0.0	0	0.0
New Mexico	0	0.0	1	15.0	1	8.0
**Total**	**112**	**9234.5**	**77**	**6157.3**	**18**	**1862.8**

Table S2: Number of Facilities and Operated capacities in the U.S. states across Q4 2024, Q4 2022, and Q1 2021.

Table S3: Callaway & Sant'Anna—Daytime Model

ATT	Std. Error	[95% Conf. Int.]
3.0439	1.4801	[0.1429 , 5.9448]*

Dynamic Effects:

Period	ATT	Std. Error	95% Simult. Conf. Band
-3	-2.0530	1.6511	[-6.2469 , 2.1408]
-2	-5.2789	2.0848	[-10.5743 , 0.0166]
-1	6.7457	1.9192	[1.8707 , 11.6206]*
0	1.1747	1.5233	[-2.6946 , 5.0440]
1	0.3708	1.4022	[-3.1910 , 3.9326]
2	0.3167	1.8571	[-4.4003 , 5.0337]
3	5.9440	1.4123	[2.3566 , 9.5314]*
4	6.8366	1.4894	[3.0534 , 10.6199]*
5	4.1478	1.7850	[-0.3861 , 8.6817]
6	2.5164	1.7603	[-1.9549 , 6.9876]

Signif. codes: '*' 95% confidence band does not cover 0
Control Group: Not Yet Treated

Table S4: Callaway & Sant'Anna—Nighttime Model

ATT	Std. Error	[95% Conf. Int.]
8.0916	0.8155	[6.4933 , 9.6899]*

Dynamic Effects:

Period	ATT	Std. Error	95% Simult. Conf. Band
-3	2.3897	0.7281	[0.4604 , 4.3189]*
-2	-1.2966	0.7340	[-3.2416 , 0.6484]
-1	0.5986	0.5875	[-0.9581 , 2.1552]
0	3.5087	0.9837	[0.9022 , 6.1153]*
1	7.1822	1.0157	[4.4909 , 9.8736]*
2	6.5310	1.0133	[3.8462 , 9.2158]*
3	9.1932	0.9586	[6.6534 , 11.7331]*
4	13.1201	1.1587	[10.0499 , 16.1904]*
5	9.3966	1.5351	[5.3293 , 13.4640]*
6	7.7096	1.0202	[5.0064 , 10.4128]*

Signif. codes: '*' 95% confidence band does not cover 0
Control Group: Not Yet Treated

Table S5: Sun & Abraham—Daytime Model

	Estimate	Std. Error	t value	P(> $|t|$)
ATT	2.4041	1.3085	1.8373	6.6639e-02 .
GDP	5.65e-07	1.60e-07	3.5209	4.6133e-04***
Population	-1.45e-04	3.10e-05	-4.6706	3.6758e-06***
Wind Share	-6.5977	4.8935	-1.3483	1.7806e-01
Solar Share	4.9037	8.9224	0.5496	5.8279e-01
Total Fossil	-1.46e-03	1.00e-03	-1.4593	1.4500e-01

Signif. codes: 0 '***' 0.001 '**' 0.01 '*' 0.05 '.' 0.1 ' ' 1

Table S6: Sun & Abraham—Nighttime Model

	Estimate	Std. Error	t value	P(> $|t|$)
ATT	8.3863	0.5920	14.1667	$< 2.2e\text{-}16$***
GDP	4.33e-07	1.61e-07	2.6792	7.5740e-03**
Population	-9.60e-05	3.13e-05	-3.0720	2.2188e-03**
Wind Share	1.2902	4.6994	0.2745	7.8376e-01
Solar Share	10.5546	9.4474	1.1172	2.6434e-01
Total Fossil	1.28e-04	1.09e-03	0.1179	9.0618e-01

Signif. codes: 0 '***' 0.001 '**' 0.01 '*' 0.05 '.' 0.1 ' ' 1

Table S7: Robustness: Sample Ending Q4 2023 (CS Nighttime Model)

ATT	Std. Error	[95% Conf. Int.]
8.4759	0.9790	[6.5570 , 10.3948]*

Dynamic Effects:

Period	ATT	Std. Error	95% Simult. Conf. Band
-3	1.6463	0.7954	[-0.4351 , 3.7277]
-2	0.0488	0.7863	[-2.0088 , 2.1064]
-1	0.4341	0.6862	[-1.3616 , 2.2298]
0	4.0972	1.1673	[1.0426 , 7.1517]*
1	9.2043	1.0361	[6.4930 , 11.9156]*
2	7.7922	1.1296	[4.8364 , 10.7481]*
3	9.2036	0.9775	[6.6456 , 11.7616]*
4	12.6810	1.4349	[8.9261 , 16.4358]*
5	5.4613	1.8511	[0.6173 , 10.3052]*
6	10.8920	1.9214	[5.8642 , 15.9197]*

Signif. codes: '*' 95% confidence band does not cover 0
Control Group: Not Yet Treated

Table S8: Robustness: Neighbouring Counties Excluded CS Daytime Model

ATT	Std. Error	[95% Conf. Int.]
3.2434	1.5554	[0.1948 , 6.2920]*

Dynamic Effects:

Period	ATT	Std. Error	95% Simult. Conf. Band
-3	-2.4289	1.8633	[-7.1722 , 2.3143]
-2	-5.8570	2.3816	[-11.9196 , 0.2057]
-1	7.1425	2.1533	[1.6611 , 12.6238]*
0	1.1918	1.6458	[-2.9979 , 5.3815]
1	0.2097	1.4707	[-3.5342 , 3.9536]
2	0.4644	2.0238	[-4.6875 , 5.6163]
3	6.6992	1.4434	[3.0247 , 10.3736]*
4	7.3253	1.6418	[3.1458 , 11.5047]*
5	4.2533	1.8785	[-0.5286 , 9.0353]
6	2.5600	1.9764	[-2.4710 , 7.5911]

Signif. codes: '*' 95% confidence band does not cover 0
Control Group: Not Yet Treated

Table S9: Robustness: Neighbouring Counties Excluded (CS Nighttime Model)

ATT	Std. Error	[95% Conf. Int.]
8.3160	0.7956	[6.7566 , 9.8753]*

Dynamic Effects:

Period	ATT	Std. Error	95% Simult. Conf. Band
-3	2.2686	0.8157	[0.1187 , 4.4185]*
-2	-1.7565	0.8037	[-3.8749 , 0.3619]
-1	0.5982	0.5897	[-0.9560 , 2.1523]
0	3.5174	0.9903	[0.9072 , 6.1276]*
1	7.2842	1.0544	[4.5053 , 10.0632]*
2	6.8036	1.0109	[4.1393 , 9.4679]*
3	9.6920	0.9311	[7.2378 , 12.1461]*
4	13.4845	1.1627	[10.4202 , 16.5489]*
5	9.5255	1.5338	[5.4831 , 13.5680]*
6	7.9045	1.0599	[5.1110 , 10.6980]*

Signif. codes: '*' 95% confidence band does not cover 0
Control Group: Not Yet Treated

Table S10: Robustness: Relaxed Coverage Requirement (CS Daytime Model)

ATT	Std. Error	[95% Conf. Int.]
2.9846	1.4352	[0.1716 , 5.7976]*

Dynamic Effects:

Period	ATT	Std. Error	95% Simult. Conf. Band
-3	-2.1049	1.6650	[-6.3081 , 2.0983]
-2	-4.8481	1.9537	[-9.7803 , 0.0841]
-1	6.2420	1.8608	[1.5443 , 10.9396]*
0	1.1522	1.4628	[-2.5406 , 4.8450]
1	0.4682	1.3411	[-2.9174 , 3.8539]
2	0.3154	1.7994	[-4.2271 , 4.8579]
3	5.7977	1.3850	[2.3012 , 9.2942]*
4	6.7109	1.4786	[2.9781 , 10.4436]*
5	4.0212	1.6840	[-0.2300 , 8.2724]
6	2.4265	1.7321	[-1.9463 , 6.7993]

Signif. codes: '*' 95% confidence band does not cover 0
Control Group: Not Yet Treated

Table S11: Robustness: Relaxed Coverage Requirement (CS Nighttime Model)

ATT	Std. Error	[95% Conf. Int.]
7.8981	0.7825	[6.3645 , 9.4317]*

Dynamic Effects:

Period	ATT	Std. Error	95% Simult. Conf. Band
-3	2.5062	0.6960	[0.6255 , 4.3870]*
-2	-1.4578	0.7056	[-3.3643 , 0.4487]
-1	0.6434	0.5609	[-0.8721 , 2.1590]
0	3.5812	0.9340	[1.0576 , 6.1049]*
1	6.8173	1.0127	[4.0810 , 9.5537]*
2	6.2940	0.9579	[3.7056 , 8.8825]*
3	9.0702	0.9218	[6.5795 , 11.5610]*
4	12.8797	1.2147	[9.5975 , 16.1620]*
5	9.1092	1.4896	[5.0842 , 13.1343]*
6	7.5347	1.0326	[4.7444 , 10.3250]*

Signif. codes: '*' 95% confidence band does not cover 0
Control Group: Not Yet Treated
